# Florivory Modulates the Seed Number-Seed Weight Relationship in *Halenia elliptica* (Gentianaceae)

**DOI:** 10.1155/2015/610735

**Published:** 2015-10-01

**Authors:** Linlin Wang, Lihua Meng, Jian Luo

**Affiliations:** ^1^Key Laboratory for Plant Diversity and Biogeography of East Asia, Kunming Institute of Botany, Chinese Academy of Sciences, Kunming 650201, China; ^2^Plant Germplasm and Genomics Center, Germplasm Bank of Wild Species, Kunming Institute of Botany, Chinese Academy of Sciences, Kunming 650201, China; ^3^Institute of Tibetan Plateau Research at Kunming, Kunming Institute of Botany, Chinese Academy of Sciences, Kunming 650201, China; ^4^Engineering Research Center of Sustainable Development and Utilization of Biomass Energy Ministry of Education, Key Laboratory of Biomass Energy and Environment Biological Technology in Yunnan Province, School of Life Sciences, Yunnan Normal University, Kunming 650500, China; ^5^Research Institute of Plateau Ecology, Tibet Agriculture and Animal Husbandry College, Linzhi, Tibet 860000, China

## Abstract

Generally, plant reproductive success might be affected negatively by florivory, and the effects may vary depending on the timing and intensity of florivory. To clarify the impacts of florivory by the sawfly larvae (Tenthredinidae) on seed production of *Halenia elliptica* D. Don, we simulated florivory by removing different proportion of flowers at three reproductive stages in this alpine herb and then examined the seed number per fruit, the seed weight, and the seed mass per fruit of the remaining flowers. Seed number per fruit reduced significantly when flowers were removed at flowering and fruiting stages or when 15% and 60% of flowers were removed. However, seed weight increased significantly after flowers were removed, independent of treatments of reproductive stage and proportion. There was a similar seed mass per fruit between the plants subjected to simulation of florivory and control. The results indicated that florivory modulated the seed number-seed weight relationship in this alpine species. Our study suggested that selective seed abortion and resource reallocation within fruits may ensure fewer but larger seeds, which were expected to be adaptive in the harsh environments.

## 1. Introduction

Herbivores include folivores, florivores, nectar robbers, seed predators, and underground root feeders [1]. Among them, florivores, the consumption of flowers, and other reproductive tissues prior to seed set [2] involve complete removal of flowers or partial damage to reproductive tissues, such as anthers, pistils, or ovaries, and can affect plant reproductive success directly and indirectly [3]. Firstly, florivores can cause total failure of seed set in some plant populations through total removal of flowers or developing seeds [4–6]. In addition, florivores can destroy accessory reproductive tissues, thereby causing reductions in male and/or female fitness either directly as a result of the physiological costs of damage [7, 8] or indirectly through decreasing pollinator service [9–13].

Florivores could be caused by larger feeders, mainly vertebrate grazers [14, 15], large beetles, orthopterans, lepidopterans, and even the tree climbing grapsid land crabs [1, 16, 17], and florivory can occur from the bud stage to seed maturation [1, 18, 19]. For example, in one species of iris, florivory at the bud stage resulted in more reduction of fruit production than that at the flower or fruiting stage [19]. In addition, the different intensity of damage can also have marked influence on the seed production [18, 20, 21]. Though full compensation occurred when 33% of a plant's flowers were removed, successive clippings that removed 55% of each plant's flowers resulted in a half decrease in seed production in* Sanicula arctopoides* [18]. Thus, florivores could affect plant reproductive success differently depending on the timing and intensity of the damage. However, the coeffects of timing and intensity of florivory were rarely investigated, which can be achieved by simulation of florivory by controlling the florivory timing and intensity [22, 23].

On the other hand, plants might compensate for herbivory damage, but the compensatory ability is different according to the time, intensity, and frequency of herbivory [18, 19, 23]. Previous studies have found that destroyed plants could compensate for their loss of flowers by producing new inflorescences [24, 25] or flowers [26, 27] or by increasing seed weight [28] and/or seed set [18, 29–31] of the remaining flowers, depending on the degree of matching between pollinators and flowering time [24]. In the present study, we aimed to examine the effects of florivory on female reproductive success of* Halenia elliptica* D. Don, ca 30% of whose flowers were destroyed by undescribed sawfly larvae in the study site, by simulating florivory at different reproductive stages under different intensity of the plants. Specifically, our objectives were to (1) evaluate the effects of florivory time and intensity on the seed number per fruit, the seed weight, and the seed mass per fruit on the plants and (2) examine the interaction of time and intensity of florivory on the female fitness of* H. elliptica*.

## 2. Material and Methods

### 2.1. Study Sites

Our field observations and experiments were carried out at the Southeast Tibet Observation and Research Station for the Alpine Environment, Chinese Academy of Sciences, from early August to the middle of October in 2012. This field station is located on the Southeast Qinghai-Tibet Plateau (latitude 29°10′–30°15′N, longitude 93°12′–95°35′E and altitude 3370 m), where the climate is characterized by a subhumid climate caused by the South Asian monsoon through the valley of the Yarlung Zangbo River, resulting in abundant summer rainfall. The average annual rainfall of the field station is 680 mm from 1961 to 2006, ca 80% of which occurs from June to September [32].

### 2.2. Study Species

**Figure 1:**
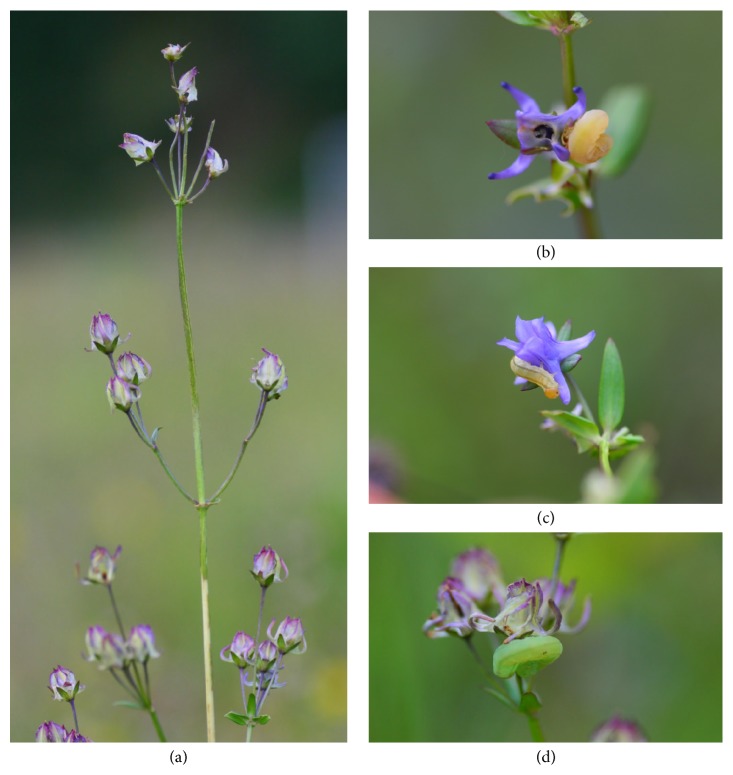
*Halenia elliptica*, floral herbivory, and florivores. (a) Floral herbivory in developing fruit stage in the whole plant; (b) floral herbivory consuming flower bud; (c) floral herbivory consuming flower; (d) floral herbivory consuming developing fruit.

*H. elliptica* is a biennial herb of the Gentianaceae. It was considered to consist of two varieties in light of the differences of flower size [33], but our previous investigations did not support the previous taxonomic treatment splitting into two varieties [34].* H. elliptica* usually grows in temperate habitats and is widely distributed in west and north China [33]. Plants of* H. elliptica* produce many flowers, and the top-positioned flowers generally open first. The flowering season is from late July to the middle of September in our study area. The corolla is blue or purple and forms four tubes with a narrow opening on the bottom and nectar is produced in spurs. This probably helps reward only specialized long-tongued pollinators [35]. Flowers were mainly visited by bumblebees in this study site, but this species was capable of setting seeds via autonomous selfing (unpublished data). Our preliminary observations found that ca 30% of* H. elliptica* flowers (27.7 ± 20.3%, mean ± SD, *n* = 100) at our study site were destroyed in the period from the bud stage to fruit maturation (Figure 1).

### 2.3. Experimental Design

The effects of floral herbivores on female reproductive success were studied by manually cutting flowers at different stages and intensity. On 4th of August, we randomly labeled 200 plants on which all flowers were in bud stage and examined the number of flowers to calculate the number of flowers to be removed from each plant. Plant heights were measured as an indicator of plant size, because florivory increased with plant size [36]. Reproductive stages were divided into bud (from bud formation to flower opening), flowering (from flower opening to wilting of petals), and fruit stages (from wilting of petals to seed dispersion). For each stage, in order to simulate different florivory intensity, different proportion of flowers (15%, 30%, and 60% of the total flowers per plant) were removed. Using the method of successive removal experiments [18], we randomly removed flowers every three days from the flower bud to seed maturity until all treatments reached the corresponding intensity. Each treatment was achieved on 20 plants using fine tweezers, and the remaining 20 plants were treated as control. Some plants were destroyed before the completion of the experiment, and so the sample size for some treatments was reduced.

In early October 2012, when the majority of fruits were mature but before dehiscence, we collected six fruits on each plant, three of which were on the top positions of the plant and the other three fruits were on the bottom of the plant, to eliminate the effects of position on seed number, because we found a significant difference (*t* = 8.66, *P* < 0.001) between the ovule numbers of upper (18.10 ± 2.92, *n* = 20) and lower part of flowers (11.85 ± 2.30, *n* = 20). We also measured the weight of natural drying seeds collected from the six fruits using a Mettler Toledo XS205 digital balance (minimum to 0.1** **mg) to estimate seed weight and seed mass per fruit.

### 2.4. Data Analysis

One-way ANOVA were used to compare the differences among plants subjected to different treatments, and General Linear Model was employed to examine the effects of florivory intensity and reproductive stage on the seed number per fruit, the seed weight, and the seed mass per fruit. All the analyses were performed with SPSS 16.

## 3. Results

No significant difference was found in the plant height among different treatments (*F*_9,184_ = 1.44, *P* = 0.17), so we did not consider the effect of plant size to female fitness in the following analysis.

### 3.1. Effects of Florivory on the Seed Number per Fruit

**Figure 2:**
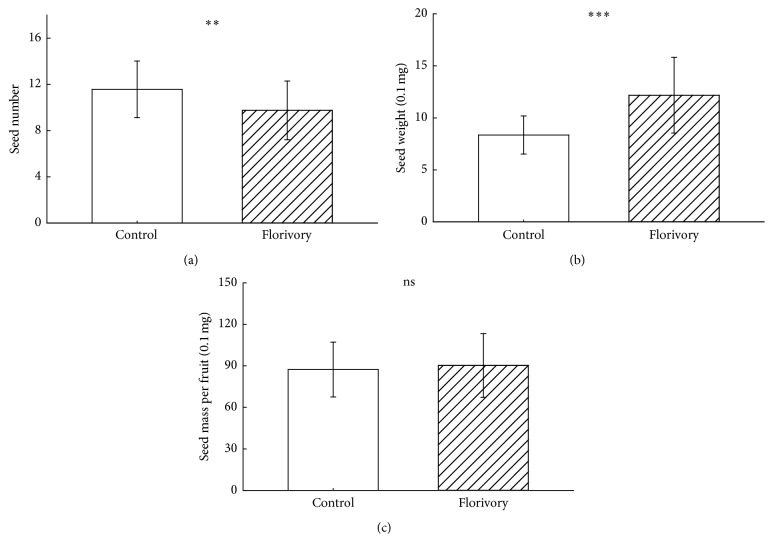
Effects of florivory on the seed number per fruit, the seed weight, and the seed mass per fruit. ^*∗∗*^ *P* < 0.01; ^*∗∗∗*^ *P* < 0.001; ns: not significant.

**Figure 3:**
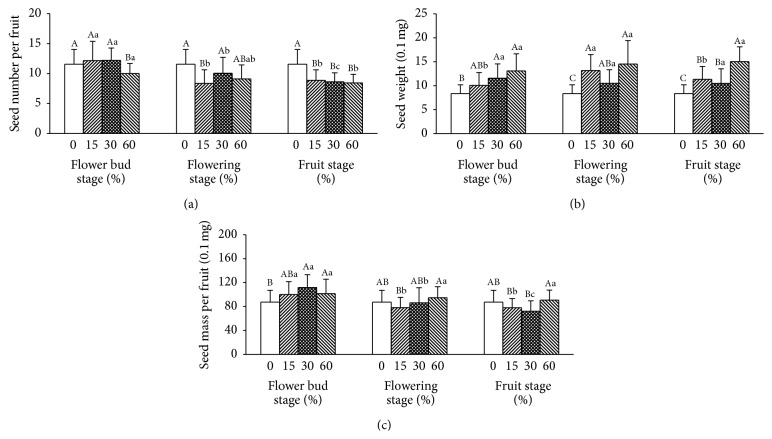
Effects of intensity of florivory (15%, 30%, 60%) at three reproductive stages on the seed number per fruit, the seed weight, and the seed mass per fruit. Data are means ± 1 SD. Uppercases above bars represent comparisons of the same reproductive stage at which florivory occurs. Lowercases represent comparisons of the same intensity of florivory. Bars with the same letters are not statistically different from each other (LSD test, 5% level).

Overall, plants subjected to simulation of florivory produced fewer seeds than the natural individuals (Figure 2(a)), and seed number was generally affected by the reproductive stages of simulating florivory, the intensity of florivory, and their interaction (Table 1). Compared with control (*n* = 19, 11.57 ± 2.45), fewer seeds were produced when the simulated florivory was carried out in flowering (*n* = 57; 9.19 ± 2.48, *F* = 13.101, *P* = 0.001) and developing fruit stages (*n* = 60; 8.65 ± 1.56, *F* = 37.751, *P* < 0.001), but seed number did not change significantly when florivory was carried out in bud stage (*n* = 58; 11.44 ± 2.57, *F* = 0.038, *P* = 0.845) (Figure 3(a)). Similarly, seed number varied remarkably with when plants were subjected to the different intensity of florivory. In comparison with control, fewer seeds were produced when 15% (*n* = 58; 9.79 ± 2.97, *F* = 5.608, *P* = 0.02) and 60% (*n* = 58; 9.19 ± 1.91, *F* = 19.151, *P* < 0.001) of flowers were removed, whereas seed number decreased insignificantly when 60% (*n* = 59; 10.27 ± 2.55, *F* = 3.822, *P* = 0.054) of flowers were removed (Figure 3(a)).

### 3.2. Effects of Florivory on the Seed Weight

Seed weight was affected significantly by florivory intensity and the interaction of reproductive stages and florivory intensity (Table 1). Overall, plants with simulating florivory produced larger seeds than control (Figure 2(b)), independent of reproductive stage and intensity of florivory (Figure 3(b)).

### 3.3. Effects of Florivory on the Seed Mass per Fruit

Overall, plants subjected to simulation of florivory and the natural plants had similar seed mass per fruit (Figure 2(c)), but seed mass per fruit was affected by florivory time, florivory intensity, and their interaction (Table 1). Compared to control (*n* = 19, 87.36 ± 19.80), seed mass per fruit was higher when the simulated florivory was carried out in bud stage (*n* = 58; 104.45 ± 22.61, *F* = 8.662, *P* = 0.004) but not in flowering (*n* = 57; 86.32 ± 21.31, *F* = 0.035, *P* = 0.853) and developing fruit stages (*n* = 60; 80.31 ± 18.04, *F* = 2.102, *P* = 0.151) (Figure 3(c)). Seed mass per fruit varied insignificantly when plants were subjected to the intensity of florivory. Fruits had similar weight when 15% (*n* = 58; 85.33 ± 20.62; *F* = 0.141, *P* = 0.708), 30% (*n* = 59; 89.70 ± 26.79; *F* = 0.123, *P* = 0.726), and 60% (*n* = 58; 95.79 ± 20.18; *F* = 2.52, *P* = 0.117) of flowers were removed from each plant (Figure 3(c)).

## 4. Discussion

Florivory can have a huge impact on resource allocation within fruits. In this study, variations of seed number and seed weight within fruits of* H. elliptica* were significant after plants were subjected to different florivory intensity at different time. The results showed that seed number per fruit decreased with increasing time and intensity of florivory, whereas seed weight increased when florivory occurred. There was a similar seed mass per fruit between the plants with simulating florivory and the natural plants. The results indicate that time and intensity of florivory has striking influences on the relationship between seed number and seed weight within fruit.

Why did florivory have different effects on seed number and seed weight at different reproductive stages and intensity? The differences can possibly be explained due to the following reasons. First, competition for resources among different plant modules has different when florivory occurred at different reproductive stages. Before fertilization, competition among genetically similar plant modules may often be involved in resource allocation, but only it can enhance overall plant fitness. After fertilization, competition for resources among genetically different plant modules has occurred through evolution by genomic conflicts between parent and offspring [37, 38]. As a result, our data revealed that seed number did not change significantly when flowers were removed in bud stage but reduced significantly when flowers were removed in flowering and developing fruit stages (Figure 3(a)), though some species aborted fewer ovaries or decreased the rate of fruit abortion when florivory occurred [31, 39]. Second, the difference in the effects of florivory would be a result of the difference in the intensity of consumption. In fact, florivory at the bud and fruit stages both tended to have negative effects on fruit production in* Iris gracilipes*, because in the bud and fruit stages severe florivory was more common than in flowering stage [19]. In addition, seed weight increased significantly when flowers were removed, irrespective of flower stage and proportion (Figure 2(b)). The interaction of time and intensity of florivory was significant on female reproductive success, indicating that the difference of the effects of florivory was caused by differences in both reproductive stage and intensity of florivory [18, 40]. Therefore,* H. elliptica* may adopt a compensation mechanism that allows resources to be shunted to later surviving flowers when florivory occurs at bud stage and modulates resource allocation within fruit after fertilization and then selective seed abortion within fruit and resource reallocation among siblings ensured fewer but higher quality seed with increasing the intensity and time of florivory, if resources allocated to flowers are limited.

It is obvious that florivory should reduce the number of open flowers, which could reduce seed number through decreasing pollinator visits [10, 12]. But bumblebee is very effective pollinator, especially in alpine environment, our preliminary observation found that the flowers of* H. elliptica* were mainly visited by bumblebees in this study site, and this species was capable of setting seeds via autonomous selfing (unpublished data) and increased florivory led to an increase in selfing by autogamy pathway [41]. These results indicate that the seed number of* H. elliptica* at our study site was limited by availability of resources rather than by reduction of pollination.

Although many studies have examined the effects of florivory on female reproductive success, few of them have distinguished the effects of the timing and intensity of florivory on the relation between seed number and seed weight within fruit [3, 9, 10, 19]. In this paper, our results showed that the relative importance of simulated florivory on seed number and weight was different depending on reproductive stages and intensity when herbivory occurred. Our results also demonstrated that seed number reduced with increasing intensity of florivory via selective seed abortion under resource limitation [42]. Under stressful environments, such as the Qinghai-Tibet Plateau, larger seeds had higher female fitness than smaller ones [43]. Similarly,* H. elliptica* yielded fewer but larger seeds in the Qinghai-Tibet Plateau when florivory occurred, indicating that female fitness may increase with increasing seed weight in this species. Our research reveals complex interactions between the intensity and time of florivory and female reproductive success.

## Figures and Tables

**Table 1:** Effects of the reproductive stages (*S*: flower bud, flowering, and developing fruit) and the intensity of florivory (*I*: 15%, 30%, 60%) on the seed number per fruit, the seed weight, and the seed mass per fruit.

	df	Seed number per fruit		Seed weight		Seed mass per fruit	
		*F*	*P*	*F*	*P*	*F*	*P*
*S*	2	27.337	<0.001	1.960	0.144	23.293	<0.001
*I*	2	3.769	0.025	18.419	<0.001	3.878	0.022
*S* × *I*	4	2.927	0.022	2.740	0.030	2.970	0.021
